# The N-terminal domain of apolipoprotein B-100: structural characterization by homology modeling

**DOI:** 10.1186/1471-2091-8-12

**Published:** 2007-07-22

**Authors:** Hassan Al-Ali, Hassan M Khachfe

**Affiliations:** 1 Computational Sciences and Bioinformatics Laboratory, American University of Beirut, Beirut, Lebanon; 2 Department of Physiology, Faculty of Medicine and Medical Center, American University of Beirut, Beirut, Lebanon

## Abstract

**Background:**

Apolipoprotein B-100 (apo B-100) stands as one of the largest proteins in humans. Its large size of 4536 amino acids hampers the production of X-ray diffraction quality crystals and hinders in-solution NMR analysis, and thus necessitates a domain-based approach for the structural characterization of the multi-domain full-length apo B.

**Results:**

The structure of apo B-17 (the N-terminal 17% of apolipoprotein B-100) was predicted by homology modeling based on the structure of the N-terminal domain of lipovitellin (LV), a protein that shares not only sequence similarity with B17, but also a functional aspect of lipid binding and transport. The model structure was first induced to accommodate the six disulfide bonds found in that region, and then optimized using simulated annealing.

**Conclusion:**

The content of secondary structural elements in this model structure correlates well with the reported data from other biophysical probes. The overall topology of the model conforms with the structural outline corresponding to the apo B-17 domain as seen in the EM representation of the complete LDL structure.

## Background

Atherosclerosis is a complex disease that has been linked to many risk factors, including hyperlipidemia, dyslipidemia, high blood pressure, and endothelial dysfunction [1]. Oxidative modification to the small low-density lipoprotein (LDL) has been dubbed the central event that initiates and propagates coronary artery diseases [2,3], and therefore, LDL is considered a major risk factor for atherosclerosis [4]. It was also shown that systemic inflammatory mechanisms may underlie the pathogenesis of atherosclerosis [5-7]. However, the specific structural interactions implicated in these mechanisms have not yet been elucidated.

Apolipoprotein B-100 (apo B) is the sole protein component of LDL [8]; however, its large size (4536 a.a.) and the limitation of current experimental techniques require that the structures of its multiple domains be analyzed separately [9,10]. Biochemical [10], calorimetric [11], computational [12-15], and spectroscopic [16] approaches were used to probe the domain arrangement and characterization of the protein, but no molecular structure has ever been assigned to any of the different domains. These techniques, however, helped in the understanding of the overall arrangement of apo B on the LDL particle and the interactions that the various secondary structures have with both the lipid and aqueous phases, and in the ability to genetically engineer protein truncations that correspond to these various domains [17-20].

In this report, we describe a model structure for apo B-17 that was modeled by homology, taking the crystal structure of lipovitellin (LV) [21-23] as a template. LV – coded 1LSH in the Protein Data Bank (PDB) repository – shares more than 30% sequence similarity with the first 782 a.a. of apo B (the N-terminal 17% of the full-length sequence), a region that is rich in disulfide bonds [24,25], essential for the secretion of the protein from hepatic cells [17], and behaves like an independent globular protein [19,20]. It seemed logical to try to characterize the structure of B17 using homology modeling as a starting step towards the study of the whole structure of apo B-100.

## Results and discussion

LDL has been termed as the *agent provocateur *of atherosclerosis. Since ApoB-100 is the sole protein component of LDL, it is expected that it plays an important role in the atherogeneity of the lipid particle. The huge size of the polypeptide hinders standard structural characterization approaches, and necessitates that it be studied in pieces, possibly correlating with the domain organization previously characterized by biochemical studies.

**Figure 1:**
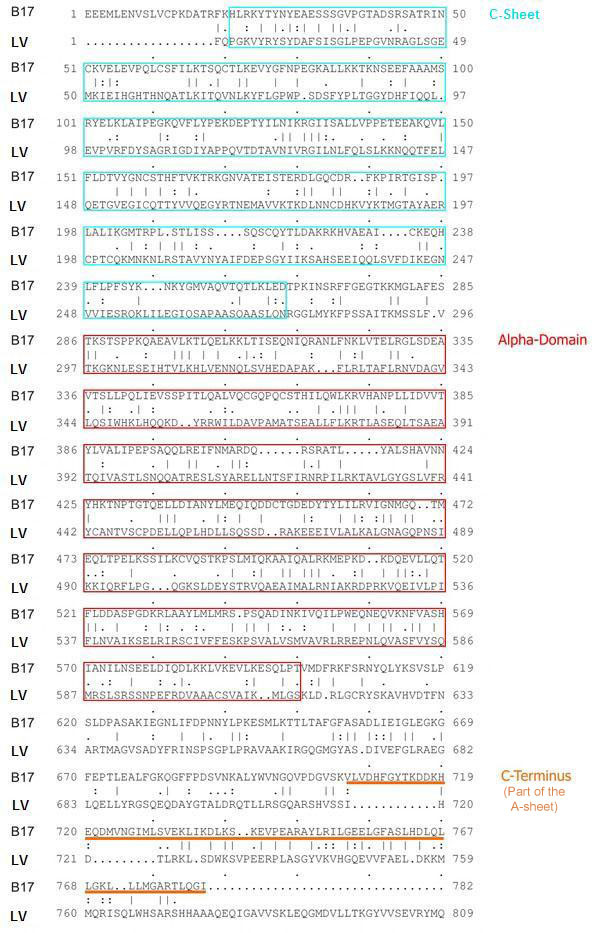
**The sequence alignment of B17 with the N-terminus of LV**. The figure also shows the different structural domains (Cyan boxes are for the βC-sheet and the magenta boxes are for the α-domain. The underlined stretch indicates the region of no electron density in the template structure.

We present here a comprehensive model structure for the N-terminal domain of apolipoprotein B-100 based on the lipovitellin crystal structure. LV, an egg yolk lipoprotein, has four β-sheet domains, labeled according to their sequence order as βC, βA, βB, and βD, and one α-helical domain labeled α, situated between the C- and A-sheets [21-23]. Lipids are transported in a pocket bounded by the three sheets βB, βA, and βD. The interaction between lipids and the other two domains, the C-sheet and the α-domain, is absent and minimal, respectively. Several rounds of multiple sequence alignments revealed that the sequence of B17 aligns best with the N-terminal C-sheet, the α-domain and a part of the A-sheet of LV (Figure 1), with about 25% sequence identity registered and an additional 15% of residue similarity obtained.

**Figure 2:**
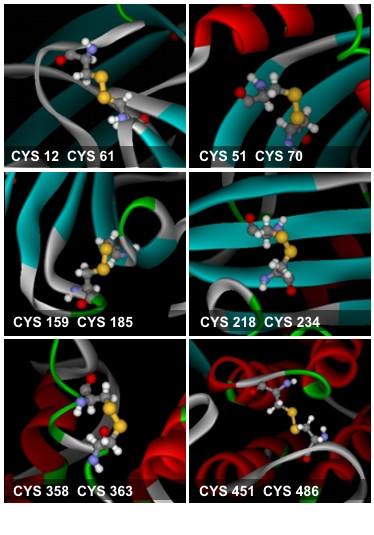
**The six disulfide bonds found in B17**. The cysteine residues are rendered in ball-and-stick representation. The sulfur atoms are colored in yellow.

Among the six disulfide bonds identified in B17 [24,25], only one pair of disulfide bonded cysteins is conserved in LV, and therefore, it was a challenge to see if the other pairs fall – or can be made to fall – within bonding proximities without steric hindrances. Indeed, the first model had the sulfur atoms of three pairs of neighboring cysteins less than 4 Å apart, whereas the rest of the cystein pairs had their sulfur atoms 5–10 Å away. To bond these latter ones, the cysteine residues were brought to bonding distances (4 Å) through a series of step wise automated or directed energy minimization (Figure 2).

One disulfide pair came within binding distance after a series of minimization runs, and two pairs approached binding distance through directed (constrained) minimization, adding up to 6 disulfide bridges. However, those that were subject to constrained minimization were located within flexible loops at the surface of the protein, and thus did not cause the overall fold to change. Minimization was done in a step-wise fashion in order to explore bonding space between the sulfur groups without distorting secondary formations. Finally, a molecular dynamics simulation at 25–27 degrees Celsius was performed on the B17 molecule to allow its side chains to explore allowed conformational space.

**Figure 3:**
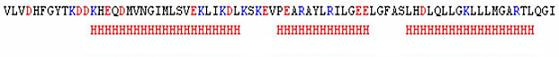
**The secondary structure prediction of the unstructured section**. The secondary structure of this C-terminus of B17 was separately modeled, and the results from PredictProtein are shown, where the H's underneath the sequence indicate helical regions.

**Figure 4:**
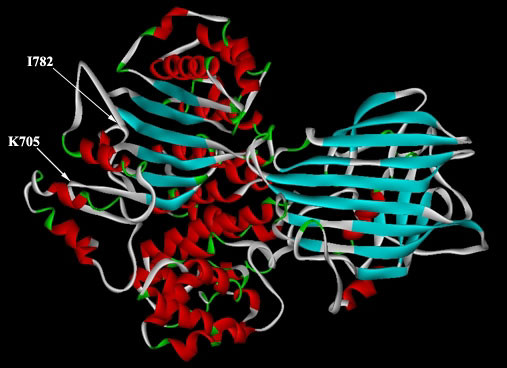
**The structure of B17 modeled after the N-terminus of LV**. β-strands are colored blue, whereas α-helices are colored red. Arrows and labels indicate the beginning and end of the 78-aa stretch modeled independent of the LV structure.

A 78-residue stretch in the A-sheet of LV has no resolving electron density, and therefore, no coordinate assignments in the crystal structure. The correspondingly aligned amino acids in B17 (residues 706 – 782) had to be modeled separately. The secondary structures of this stretch were predicted using a variety of algorithms, including the Chou-Fasman algorithm [26], the PROF methods of PredictProtein [27,28], and the SPDBV modality of Deep View. All of these modalities suggested an-all-helical structure of the stretch, with a helical content around 65 % (Figure 3) and a reliability index approaching 90%. Several rounds of energy minimization and simulation – first in vacuum and later in water as a solvent – were performed allowing the previously-unstructured region to adopt a stable fold while its ends were fixed in space at coordinates corresponding to the crystal structure amino acids immediately preceding and succeeding the beginning and end residues in the primary sequence, respectively. Then, using the LIGATE modality in HOMOLOGY, the structure of this part was pinned to the corresponding extremities in the LV-modeled B17, and the energy of the whole molecule was minimized again (Figure 4).

It should be noted here that a well-ordered structure is to be expected in this stretch owing to the fact that earlier biophysical studies suggested the presence of secondary structural elements that cannot be accounted for by what is reported in the crystal structure of LV only [19,20]. The secondary structural content in this complete model correlates excellently with the data reported previously using those biophysical probes. The structure also confirms the exposure of several coil-bound histidine residues that may be implicated in some helical rearrangement upon their protonation due to a slight decrease in the solvent pH [19,20]. The accessibility of these residues to the aqueous solvent was tested (Table 1), and their protonation upon the decrease in pH was confirmed.

**Table 1:** Solvent accessibility for the buried salt bridge.

		**Solvent Accessibility Surface Area (Å^2^)**	
		
**Residue**	**Atom**	**Before Solvation**	**After Solvation**
K530	NZ	75.20	0.89
E557	OE2	1.54	0.00

Solvent accessibility surface areas for individual atoms calculated according to the hydration states of the corresponding atoms

The structure of LV has been reported to contain a completely buried salt bridge formed between R547 and E574 [21], which ties together the two "helical sheets" in the α-domain, thereby increasing the stability of the local fold. A careful inspection of the B17 model structure revealed that a very similar salt bridge is formed between K530 and E557, which align – sequentially – with the above-mentioned residues in LV. Moreover, the solvent accessibility analysis illustrates that the involved side chains are well shielded form the aqueous medium and can therefore account for an extra stability in the α-domain of B17 that has been previously reported [19,20].

**Figure 5:**
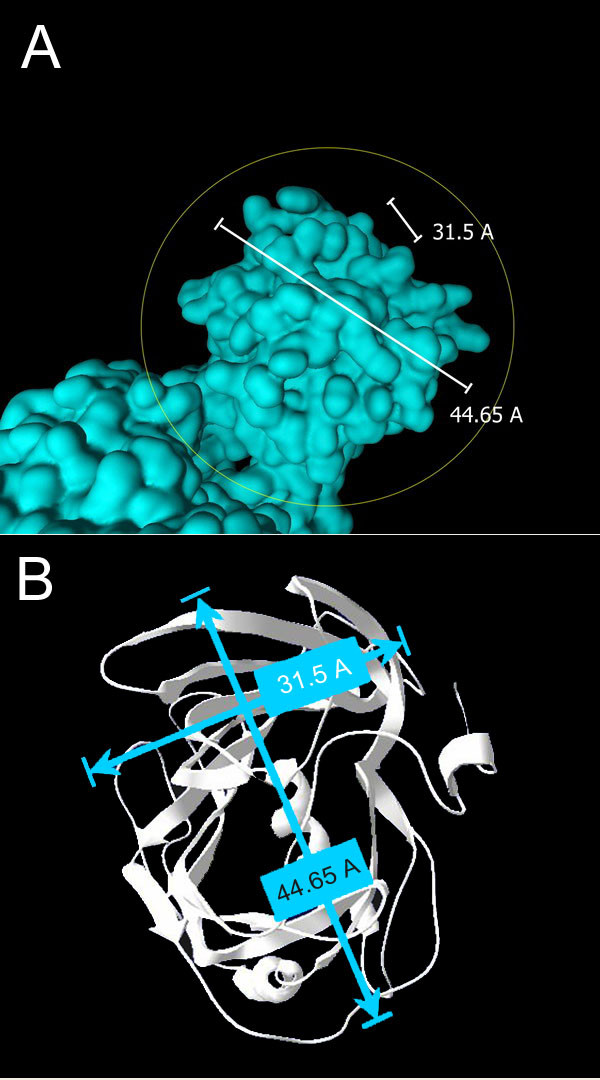
**The spatial dimensions of the β-domain of B17**. (A) Side view and (B) top view of the N-terminus of B17 with the corresponding dimensional values. These values correlate perfectly with the "knob" structure seen on the LDL particle and mapped to the N-terminus of B100.

Electron microscopy studies of intact LDL particles [29,30] showed that the N-terminus of apo B has a knob-shaped electron density with dimensions 30 – 45 Å. These dimensions approximate perfectly with the β-domain in the B17 model (Figure 5). These dimensions, along with the positions of the disulfide bonds and the buried, conserved salt bridge in the helical region, give credibility to the model. The lipid pocket surface accessibility – for potential lipid recruitment – towards the inside of the α-domain also makes the structure trustworthy.

**Figure 6:**
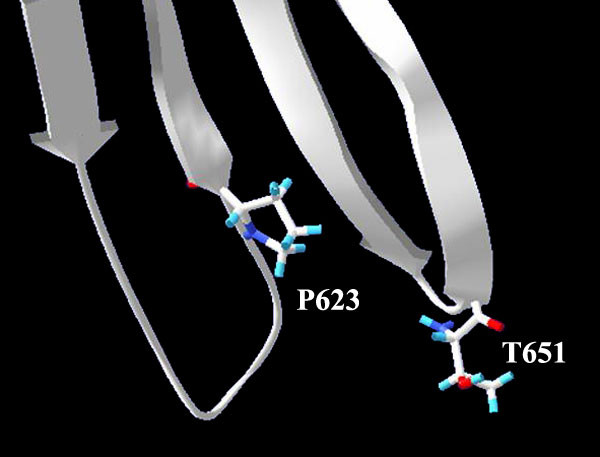
**Fold versus angle stability: **P623 and T651, with Z-scores around -3.0, fall in turns involved in a mini sheet between the helical region and the C-terminus. The elevated torsion energy is compensated by an overall fold energy reduction.

**Figure 7:**
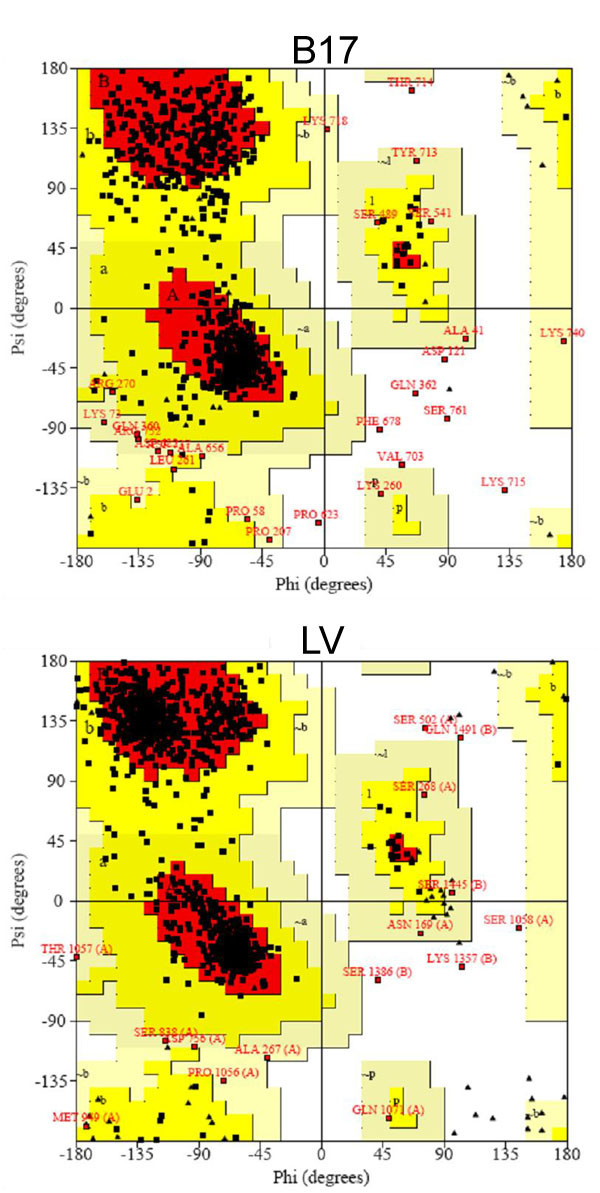
**The Ramachandran plots of B17 and LV**. More than 97% of the backbone angle-pairs in B17 fall within the favorable regions, whereas 2.3% fall within the additionally allowed regions, and less than 1% is in the disallowed regions. This is very similar to the data from the LV system (98% in the favorable regions, 1% in the additionally allowed regions, and 1% in the disallowed regions).

A comprehensive structure validation test was carried out to check the physical elements of the model. Bond angles were found to deviate normally from the reported mean standard values [31]. Moreover, the RMS Z-score for bond angles in this model structure is within 9 % change with respect to that in the template structure. Bond lengths were found to have normal variability. The contact distances of all atom pairs have been checked. Among the 31 reported abnormally short interatomic distances in B17 (more than 200 in the corresponding LV template), 23 are either representations of hydrogen bonds or predictions of atoms with B-factors higher than 80, indicating that the atoms potentially implicated in these bumps are not there anyway. The evaluation of the model torsion angles did indeed show some unusual residues; however, the two amino acids, P623 and T651, with Z-scores around -3.0 (the worrying limit), actually fall in the region joining the α-domain with the C-terminus of the protein. P623 is at the end of a β-strand and T651 is the second of a two-residue turn between two strands as well, all three of these strands are involved in a mini sheet between the helical region and the C-terminus (Figure 6), and, therefore, the slight increase in their torsional energy is compensated by the overall fold stability. Finally, the Ramachandran plot of the backbone psi-phi angles of the B17 model showed comparative results to those obtained from the crystal structure of LV (Figure 7).

## Conclusion

This model provides further insight into the structural basis for the functional attributes of B-17, and constitutes a step towards the full elucidation of the multi-domain structure of full-length Apo B-100. While the current structure ensures the globular topology of the domain and its poor lipidation state, as it does not show lipid binding pockets, the biological implications of this protein – independent of its role in apo B-100 – remain to be tested *in vitro *and, later, *in vivo*, since B17 is not a naturally occurring plasma apolipoprotein. Knowing the importance of this domain in the secretion and assembly of the full-length apo B-100, we anticipate that the current structure and subsequent physiological experiments will assist in the development of novel drugs for the treatment of and protection against diseases correlated with elevated blood LDL.

## Methods

### Molecular modeling

#### Sequence alignment

Multiple sequence alignments were done using BLAST [32] and the alignment module of the Discovery Studio suite (Accelrys Inc., Discovery Studio 1.5, San Diego: Accelrys Inc., 2004)

#### Structure prediction

The structure of B17 (residues 1–704) was modeled using MODELLER [33] of HOMOLOGY in insight II (Accelrys Inc., Insight Modeling Environment, Release 2000.1, San Diego: Accelrys Inc., 2002), based on the crystal structure of lipovitellin (LV), an egg yolk protein that shares over 30% sequence homology (in over 700 amino acid overlap) with B17. The secondary structure of the unstructured region was predicted using the Chou-Fasman Algorithm [26], the PROF methods [27,28] and the Deep View modality [34]. The calculation was performed using the Accelrys SeqWeb server of the GCG Wisconsin Package.

### Simulation

#### Energy calculations

EC's were performed using DISCOVER (Accelrys Inc., CDiscover Molecular Simulator, Release 2000.1, San Diego: Accelrys Inc., 2002) and CHARMm (Version c28b) [35] modules in Insight II. Energy minimizations were performed using the Steepest Descent method followed by Conjugate Gradients.

#### Molecular dynamics

MD Simulations were carried out with periodic boundary conditions using a cubic box (of appropriate size), in the Insight II package. Solvent water molecules were represented by the three-site TIP3P water model [36], in the NVT ensemble.

#### Force fields

Calculations were performed using the DISCOVER force-fields CVFF and CFF91. The CHARMm force-field used in the solvation simulation was CHARMm27.

### Analysis

#### Solvent accessibility

Solvent Accessible Surface Area (SASA) was calculated for individual atoms using the Structural Biology at NIH server (Structools), with a probe radius of 1.4 Å [37].

#### Potential maps

Solvation energy and hydrophobic interactions were calculated using the Delphi module in Insight II (Accelrys Inc., Delphi Module, Release 2000.1, San Diego: Accelrys Inc., 2002), using the CFF91 force-field. Potential maps were constructed using a grid. The dielectric value was assigned as 4 for the protein and 80 for the solvent.

### Structure validation

Structure validation tests were carried out using the PROCHECK [38] and WHATIF [39] modalities. Calculated values were referenced to the reported mean standard values [31].

## Authors' contributions

HAA carried out all structural prediction and optimization exercises and participated in the analysis of the results. HMK conceived of the study, designed the experimental approach, coordinated the work, analyzed the results, and drafted the manuscript. All authors read and approved the final manuscript.

## References

[B1] Soltero-Perez IF (2003). Thinking intelligently about therapy of atherosclerosis. American Journal of Therapeutics.

[B2] Yla-Herttuala S, Palinski W, Rosenfeld ME, Parthasarathy S, Carew TE, Butler S, Witztum JL, Steinberg D (1989). Evidence for the presence of oxidatively modified low density lipoprotein in atherosclerotic lesions of rabbit and man. Journal of Clinical Investigation.

[B3] Yla-Herttuala S, Palinski W, Rosenfeld ME, Steinberg D, Witztum JL (1990). Lipoproteins in normal and atherosclerotic aorta. European heart journal.

[B4] Archbold RA, Timmis AD (1999). Modification of coronary artery disease progression by cholesterol-lowering therapy: the angiographic studies. Current opinion in lipidology.

[B5] Bach-Ngohou K, Nazih H, Nazih-Sanderson F, Zair Y, Le Carrer D, Krempf M, Bard JM (2001). Negative and independent influence of apolipoprotein E on C-reactive protein (CRP) concentration in obese adults. Potential anti-inflammatory role of apoE in vivo. International Journal of Obesity & Related Metabolic Disorders: Journal of the International Association for the Study of Obesity.

[B6] Hulthe J, Fagerberg B (2002). Circulating oxidized LDL is associated with increased levels of cell-adhesion molecules in clinically healthy 58-year old men (AIR study). Medical Science Monitor.

[B7] Titov VN (2003). The functional role of arterial intima. Endogenous and exogenous pathogens and specificity of atheromatosis as an inflammation. Klinicheskaia Laboratornaia Diagnostika.

[B8] Mahley RW, Angelin B (1984). Type III hyperlipoproteinemia: recent insights into the genetic defect of familial dysbetalipoproteinemia. Advances in Internal Medicine.

[B9] Cladaras C, Hadzopoulou-Cladaras M, Nolte RT, Atkinson D, Zannis VI (1986). The complete sequence and structural analysis of human apolipoprotein B-100: relationship between apoB-100 and apoB-48 forms. EMBO Journal.

[B10] Yang CY, Gu ZW, Weng SA, Kim TW, Chen SH, Pownall HJ, Sharp PM, Liu SW, Li WH, Gotto AM (1989). Structure of apolipoprotein B-100 of human low density lipoproteins. Arteriosclerosis.

[B11] Walsh MT, Atkinson D (1990). Calorimetric and spectroscopic investigation of the unfolding of human apolipoprotein B. Journal of lipid research.

[B12] Nolte RT (1994). Structural analysis of the human apolipoproteins: An integrated approach utlilizing physical and computational methods. PhD Dissertation.

[B13] Segrest JP, Garber DW, Brouillette CG, Harvey SC, Anantharamaiah GM (1994). The amphipathic alpha helix: a multifunctional structural motif in plasma apolipoproteins. Advances in Protein Chemistry.

[B14] Segrest JP, Jones MK, Mishra VK, Pierotti V, Young SH, Boren J, Innerarity TL, Dashti N (1998). Apolipoprotein B-100: conservation of lipid-associating amphipathic secondary structural motifs in nine species of vertebrates. Journal of lipid research.

[B15] Segrest JP, Jones MK, De Loof H, Dashti N (2001). Structure of apolipoprotein B-100 in low density lipoproteins. Journal of lipid research.

[B16] Walsh MT, Atkinson D (1986). Physical properties of apoprotein Bin mixed micelles with sodium deoxycholate and in a vesicle with dimyristoyl phosphatidylcholine. Journal of lipid research.

[B17] Herscovitz H, Hadzopoulou-Cladaras M, Walsh MT, Cladaras C, Zannis VI, Small DM (1991). Expression, secretion, and lipid-binding characterization of the N-terminal 17% of apolipoprotein B. Proceedings of the National Academy of Sciences of the United States of America.

[B18] Herscovitz H, Kritis A, Talianidis I, Zanni E, Zannis V, Small DM (1995). Murine mammary-derived cells secrete the N-terminal 41% of human apolipoprotein B on high density lipoprotein-sized lipoproteins containing a triacylglycerol-rich core. Proceedings of the National Academy of Sciences of the United States of America.

[B19] Khachfe HM, Atkinson D (2001). Structural Analysis and Characterization of The 17% N-terminal Domain of Apolipoprotein B-100 Using CD Spectroscopy [abstract]. Biophys J.

[B20] Khachfe HM (2002). Spectroscopic and Calorimetric Studies of the 17% N-terminal Domain of Apolipoprotein B-100. PhD Dissertation.

[B21] Mann CJ, Anderson TA, Read J, Chester SA, Harrison GB, Kochl S, Ritchie PJ, Bradbury P, Hussain FS, Amey J, Vanloo B, Rosseneu M, Infante R, Hancock JM, Levitt DG, Banaszak LJ, Scott J, Shoulders CC (1999). The structure of vitellogenin provides a molecular model for the assembly and secretion of atherogenic lipoproteins. Journal of Molecular Biology.

[B22] Raag R, Appelt K, Xuong NH, Banaszak L (1988). Structure of the lamprey yolk lipid-protein complex lipovitellin-phosvitin at 2.8 A resolution. Journal of Molecular Biology.

[B23] Segrest JP, Jones MK, Dashti N (1999). N-terminal domain of apolipoprotein B has structural homology to lipovitellin and microsomal triglyceride transfer protein: a "lipid pocket" model for self-assembly of apob-containing lipoprotein particles. Journal of lipid research.

[B24] Shelness GS, Thornburg JT (1996). Role of intramolecular disulfide bond formation in the assembly and secretion of apolipoprotein B-100-containing lipoproteins. Journal of lipid research.

[B25] Yang CY, Kim TW, Weng SA, Lee BR, Yang ML, Gotto AM (1990). Isolation and characterization of sulfhydryl and disulfide peptides of human apolipoprotein B-100. Proceedings of the National Academy of Sciences of the United States of America.

[B26] Chou PY, Fasman GD (1974). Prediction of protein conformation. Biochemistry.

[B27] Rost B, Sander C (1993). PROF. J Mol Biol.

[B28] Rost B, Fariselli P, Casadio R (1996). PROFhtm. Prot Science.

[B29] Orlova EV, Sherman MB, Chiu W, Mowri H, Smith LC, Gotto AM (1999). Three-dimensional structure of low density lipoproteins by electron cryomicroscopy. Proceedings of the National Academy of Sciences of the United States of America.

[B30] Poulos GW (2001). The three dimensional structure of low density lipoprotein via cryoelectron microscopy. PhD Dissertation.

[B31] Engh R, Huber R (1991). Accurate Bond and Angle Parameters for X-ray Protein Structure Refinement. Acta Crystallogr.

[B32] Altschul SF, Lipman DJ (1990). Protein database searches for multiple alignments. Proc Natl Acad Sci USA.

[B33] Sali A, Potterton L, Yuan F, van Vlijmen H, Karplus M (1995). Evaluation of comparative protein modeling by MODELLER. Proteins.

[B34] Swiss-PDB viewer. http://www.expasy.org/spdbv.

[B35] Brooks BR, Bruccoleri RE, Olafson BD, States DJ, Swaminathan S (1983). CHARMM:A program for Macromolecular Energy, Minimization, and Dynamics Calculations. J Comp Chem.

[B36] Jorgensen WL, Chandrasekhar J, Buckner JK, Madura JD (1986). Computer simulations of organic reactions in solution. Annals of the New York Academy of Sciences.

[B37] Gerstein M (1992). A Resolution-Sensitive Procedure for Comparing Protein Surfaces and its Application to the Comparison of Antigen-Combining Sites. http://www.ncbi.nlm.nih.gov/structools.htm.

[B38] Laskowski RA, MacArthur MW, Moss DS, Thornton JM (1993). PROCHECK: a program to check the stereochemical quality of protein structures. J Appl Cryst.

[B39] Vriend G (1990). WHAT IF: a molecular modelling and drug design program. J Mol Graph.

